# Recombinant amelogenin regulates the bioactivity of mouse cementoblasts in vitro

**DOI:** 10.1038/s41368-018-0010-5

**Published:** 2018-05-09

**Authors:** Sema S. Hakki, S. Buket Bozkurt, Emre Türkay, Michel Dard, Nuhan Purali, Werner Götz

**Affiliations:** 1 Department of Periodontology, Faculty of Dentistry, Selçuk University, 42079 Konya, Turkey; 2 Research Center of Dental Faculty, Selçuk University, Konya, Turkey; 3 Department of Orthodontics, Oral Biology Laboratory, Faculty of Dentistry, University of Bonn, Bonn, Germany; 4 Department of Periodontology and Implant Dentistry, College of Dentistry, New York University, New York, NY USA; 5 Department of Biophysics, Faculty of Medicine, Hacettepe University, Ankara, Turkey

## Abstract

Amelogenin (AMG) is a cell adhesion molecule that has an important role in the mineralization of enamel and regulates events during dental development and root formation. The purpose of the present study was to investigate the effects of recombinant human AMG (rhAMG) on mineralized tissue-associated genes in cementoblasts. Immortalized mouse cementoblasts (OCCM-30) were treated with different concentrations (0.1, 1, 10, 100, 1000, 10,000, 100,000 ng · mL^-1^) of recombinant human AMG (rhAMG) and analyzed for proliferation, mineralization and mRNA expression of bone sialoprotein (BSP), osteocalcin (OCN), collagen type I (COL I), osteopontin (OPN), runt-related transcription factor 2 (Runx2), cementum attachment protein (CAP), and alkaline phosphatase (ALP) genes using quantitative RT-PCR. The dose response of rhAMG was evaluated using a real-time cell analyzer. Total RNA was isolated on day 3, and cell mineralization was assessed using von Kossa staining on day 8. COL I, OPN and lysosomal-associated membrane protein-1 (LAMP-1), which is a cell surface binding site for amelogenin, were evaluated using immunocytochemistry. F-actin bundles were imaged using confocal microscopy. rhAMG at a concentration of 100,000 ng · mL^-1^ increased cell proliferation after 72 h compared to the other concentrations and the untreated control group. rhAMG (100,000 ng · mL^-1^) upregulated BSP and OCN mRNA expression levels eightfold and fivefold, respectively. rhAMG at a concentration of 100,000 ng · mL^-1^ remarkably enhanced LAMP-1 staining in cementoblasts. Increased numbers of mineralized nodules were observed at concentrations of 10,000 and 100,000 ng · mL^-1^ rhAMG. The present data suggest that rhAMG is a potent regulator of gene expression in cementoblasts and support the potential application of rhAMG in therapies aimed at fast regeneration of damaged periodontal tissue.

## Regenerative medicine: Engineered amelogenin boosts oral cell activity

A protein with its roots in dental development stimulates the proliferation and gene expression of cells linked to regeneration. Amelogenin is a mediator of enamel and tooth root formation, and the main component of a recently-developed medicine for periodontal regeneration. An international research group led by Sema Hakki, of Selcuk University, Turkey, has now elucidated the effects of amelogenin on cementoblasts, a type of cell responsible for producing the vital, mineralized layer on surface of the tooth root. Hakki’s team found that the bacteria-derived amelogenin increased the rate of mouse cementoblast proliferation and mineralization in vitro, and increased the expression of genes related to bone and tissue generation. The team also demonstrated the presence of a likely amelogenin receptor on the cells used in their study. These findings support further investigation into amelogenin’s therapeutic potential.

## Introduction

Enamel matrix derivative (EMD) is a group of enamel matrix proteins that are derived from Hertwig’s root sheath of developing porcine teeth. In vitro studies have shown positive effects of EMD on the proliferation of periodontal ligament (PDL) cells, gingival fibroblasts, follicle cells, and cementoblasts.^1–7^ Commercially available EMD suspended in a hydrogel (Emdogain®, Institut Straumann AG, Basel, Switzerland) is used for regenerative therapy around natural teeth. This formulation is a specific approach for enhancing periodontal regeneration. Clinical studies have indicated that EMD treatment positively influences periodontal wound healing and regeneration in humans.^8^ Most human clinical trials and case series of Emdogain application have demonstrated significant improvements in probing depth radiographic evidence of bone augmentation and cementum regeneration alone or in combination with bone grafts.^7,9–14^ Histological results from animal and human studies have confirmed these positive effects. EMD has been shown to be osteogenic and cementogenic,^15,16^ and it exerts a direct effect on osteoblasts via the enhancement of mineralization activity.^17^ Additionally it is known that EMD can interact with various other cells, including osteoblasts and dental stem cells.^4,18–23^

EMD is a hierarchical complex of proteins, and the identity of the components responsible for its biological effects is not known. Amelogenin (AMG) is an enamel matrix protein that is secreted by ameloblasts. AMG constitutes ~90% of the extracellular matrix of enamel, and it is the primary component of EMD. AMG has an important role in the mineralization of enamel and regulates mineralized tissue-associated factors during dental development, including root formation.^24,25^ AMG is also a signaling molecule in epithelial–mesenchymal interactions during odontogenesis and root formation.^1^ AMGs were initially considered tissue specific and exclusively expressed by the enamel-producing ameloblast cells. However, various isoforms have been found in the dentin matrix and associated odontoblasts. Recent reports suggest the expression of AMG in the periodontal ligament and Hertwig’s epithelial root sheath of the developing tooth attachment apparatus and other tissues.^26^ AMG polypeptides have been associated with cell signaling and may exhibit osteogenic potential. Tompkins et al.^27^ characterized an AMG cell surface receptor in the mouse, lysosome associated membrane protein-1 (LAMP-1), which is also found at cell surfaces, where it acts as a binding protein that may be involved in the interaction between cells and AMG. Exogenously added AMG is taken up by cells into LAMP-1-positive vesicles.^28^

Recent studies revealed that AMG-derived peptides exhibited potential as a useful tool for the treatment of periodontal and orthopedic diseases.^4^ Gungormus et al. used AMG-derived peptide 5 (ADP5) as a biomineralizing protein to engineer mineral formation and promote periodontal tissue regeneration. These authors suggested that the cementomimetic (e.g., cementum-like) layer formed by ADP5 may be used clinically to repair diseased root surfaces. AMG and its peptides contribute to the cell-based regeneration of periodontal tissues. Therefore, an understanding of the AMG-mediated signaling factors that regulate cementogenesis is critical.^29^

EMD and its components, e.g., AMG, are obtained via extraction from the crude enamel matrix, but recent studies attempted recombinant technology. Simmer et al.^30^ first described recombinant AMG produced in *E. coli* using a splice variant of murine AMG. Deutsch et al.^31^ and Taylor et al.^32^ described the expression of a recombinant human AMG protein in a eukaryotic baculovirus system for the first time. Their in vivo regeneration studies revealed that the recombinant AMG protein substantially increased the regeneration of periodontal tissues after induced periodontitis.^33^ Li et al.^34^ developed a recombinant AMG, rh174, using an *E. coli* system. Svensson et al.^35^ successfully developed another process for the purification of human recombinant AMG in *E. coli* based on the solubility properties of AMG and examined its effects on osteoblasts in vitro*.*A recent clinical study suggested that rhAMG enhanced apex formation and induced connective tissue regeneration.^36^ The purpose of the present study was to investigate the effects of the rhAMG developed by Svensson et al.^35^ on the proliferation, mineralization and expression of mineralized tissue-associated genes and the cementum-specific marker cementum attachment protein (CAP) in mouse cementoblasts.

## Results

### Real-time cell analysis results

**Fig. 1:**
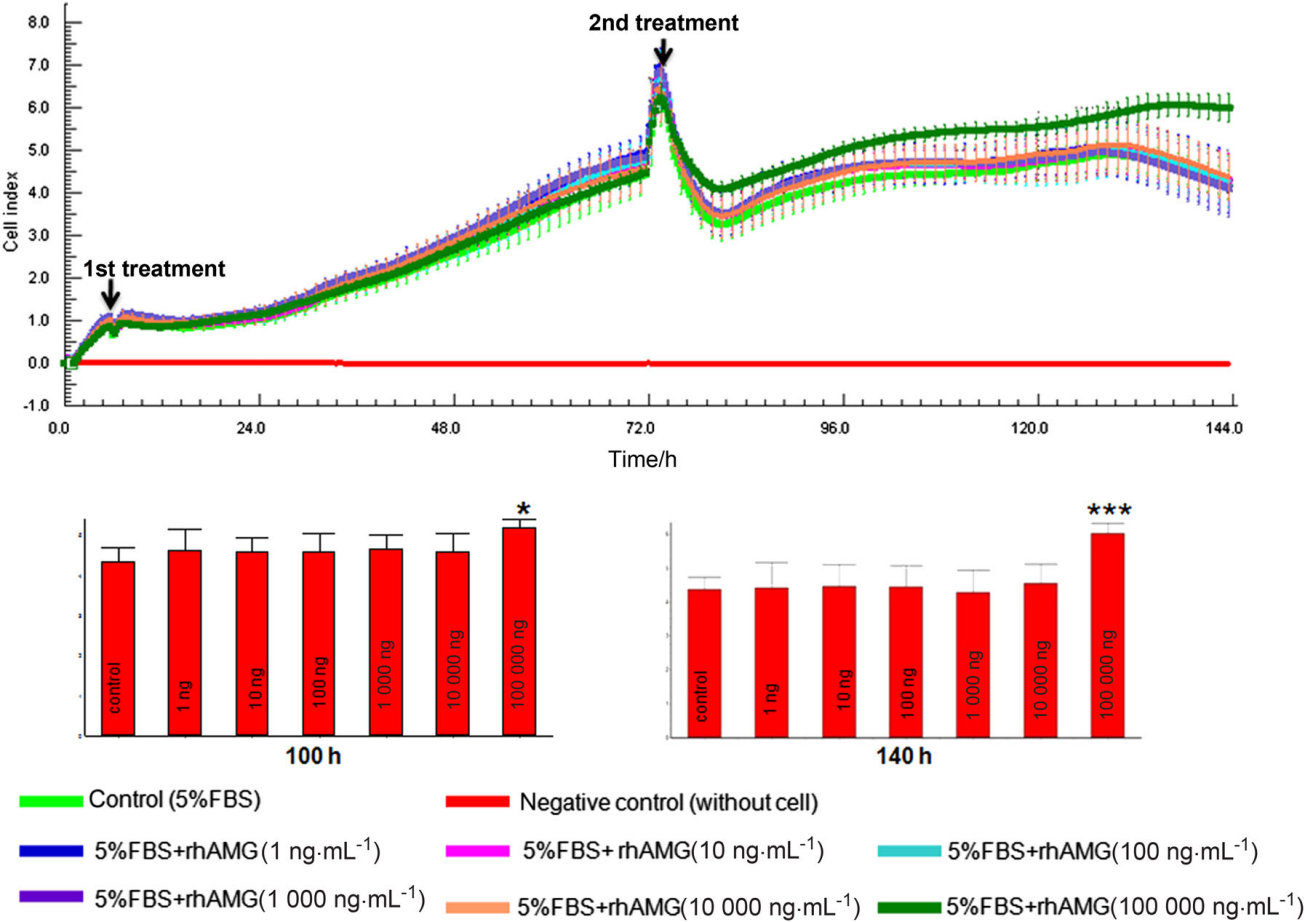
Cell index graph of OCCM-30 cells (*n*:3) treated with different concentrations of rhAMG using RTCA-SP. Cells treated with 100,000 ng · mL^-1^ exhibited a higher cell proliferation rate than other concentrations and the control group. Proliferation experiments were repeated twice. Bottom graphs show the difference between the concentrations at 100 and 140 h. rhAMG, recombinant human amelogenin

There were no significant differences among rhAMG concentrations until 72 h, when an apparent increase was noted in the 100,000 ng · mL^-1^ group (Fig. 1). Only 100,000 ng · mL^-1^ rhAMG-stimulated OCCM-30 cell proliferation, and the other concentrations of AMG exhibited no effect.

### Effects of rhAMG on the mRNA expression levels of mineralized tissue-related genes in OCCM-30 cells

**Fig. 2:**
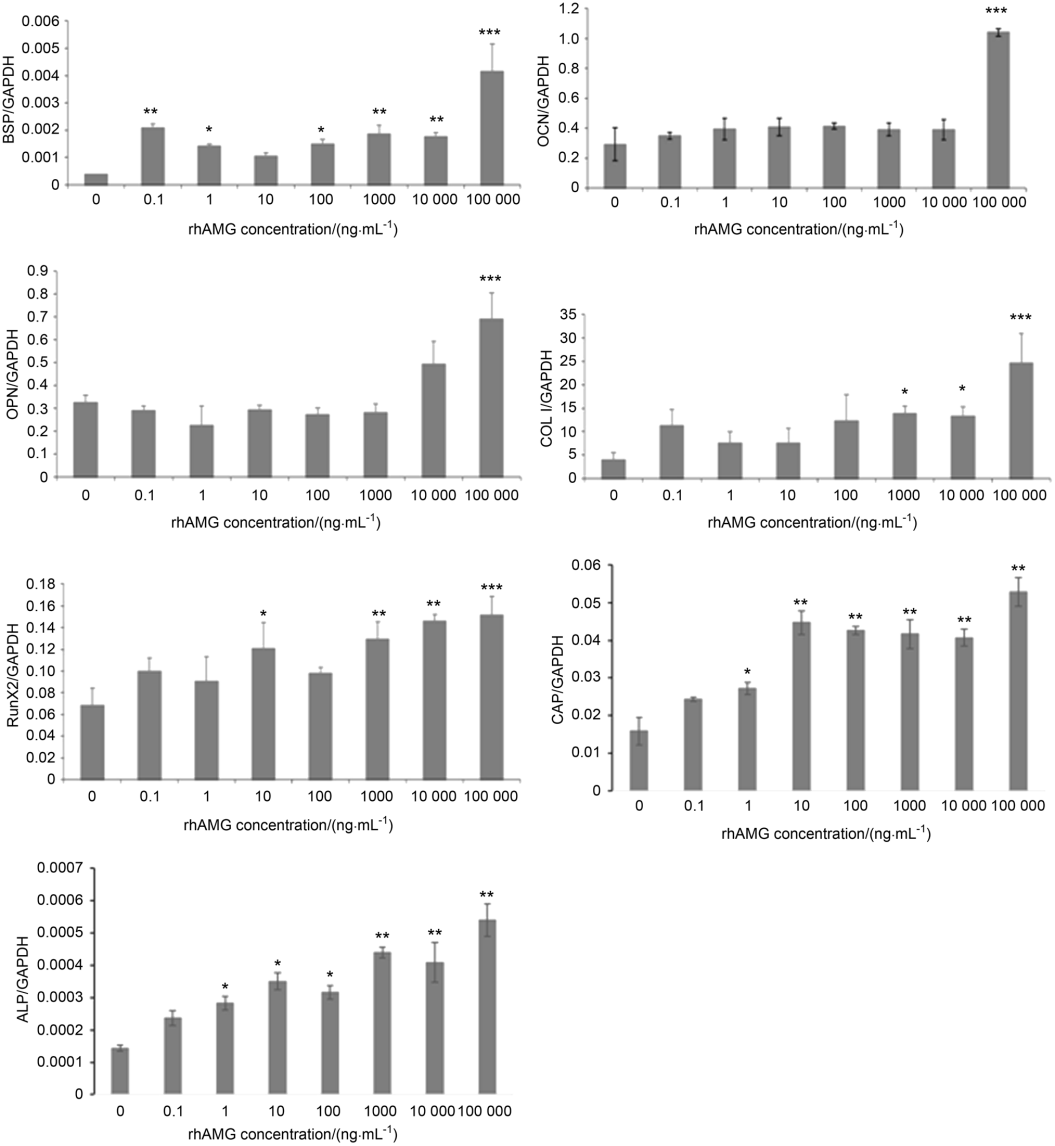
mRNA expression of mineralized tissue-associated genes (BSP, OCN, OPN, COL1, Runx2, CAP, and ALP) in OCCM-30 cells using quantitative RT-PCR. (Target genes were normalized to housekeeping genes). Experiments were repeated three times. ***P < 0.001, **P < 0.01, *P < 0.05. rhAMG, recombinant human amelogenin

As shown in Fig. 2, the mRNA expression levels of BSP in OCCM-30 cells was upregulated significantly (*P* < 0.01) after rhAMG treatment. The most pronounced upregulation (approximately eightfold) was observed in the 100,000 ng · mL^-1^ rhAMG group. RhAMG-stimulated runt-related transcription factor 2 (Runx2) mRNA levels in a dose-dependent manner. The highest rhAMG concentrations (10,000 and 100,000 ng · mL^-1^) upregulated the OPN transcript. RhAMG concentrations of 0.1–10,000 ng · mL^-1^ did not alter OCN mRNA expression, but 100,000 ng · mL^-1^ rhAMG stimulated at least a threefold increase in OCN transcription in OCCM-30 cells. COL I mRNA expression was stimulated by all concentrations of rhAMG. The most significant increase in COL I mRNA expression (approximately fivefold) was observed after 100,000 ng · mL^-1^ rhAMG treatment. ALP and CAP mRNA expression was upregulated at 1 ng · mL^-1^ rhAMG (*P* < 0.05), and a more pronounced upregulation was observed at higher concentrations (*P* < 0.01)

### Mineralization

**Fig. 3:**
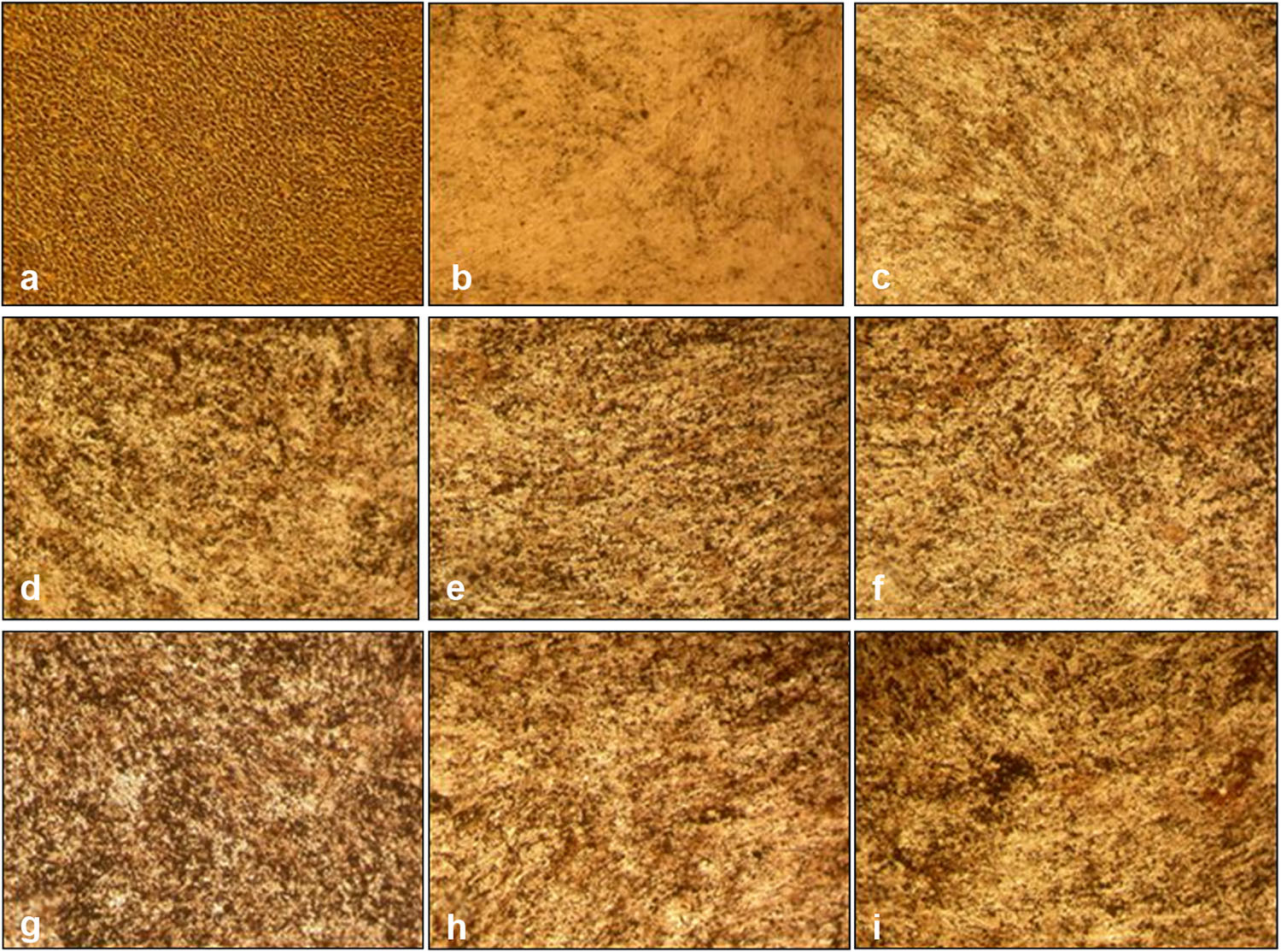
Biomineralization of OCCM-30 cells. Osteogenic differentiation was indicated by the formation of calcified nodules with von Kossa staining on day 8. **a** negative control (without mineralization media), **b** positive control [with mineralization media: ascorbic acid (AA, 50 μg/ml) and β-glycerophosphate (BGP, 10 mM)], **c** 0.1 ng · mL^-1^, **d** 1 ng · mL^-1^, **e** 10 ng · mL^-1^, **f** 100 ng · mL^-1^, **g**1000 ng · mL^-1^, **h** 10.000 ng · mL^-1^, **i** 100.000 ng · mL^-1^

All concentrations of rhAMG resulted in increased biomineralization (Fig. 3). This induction was more apparent in groups treated with 10,000 and 100,000 ng · mL^-1^ rhAMG than in the untreated groups.

### Immunocytochemistry

#### Osteopontin

**Fig. 4:**
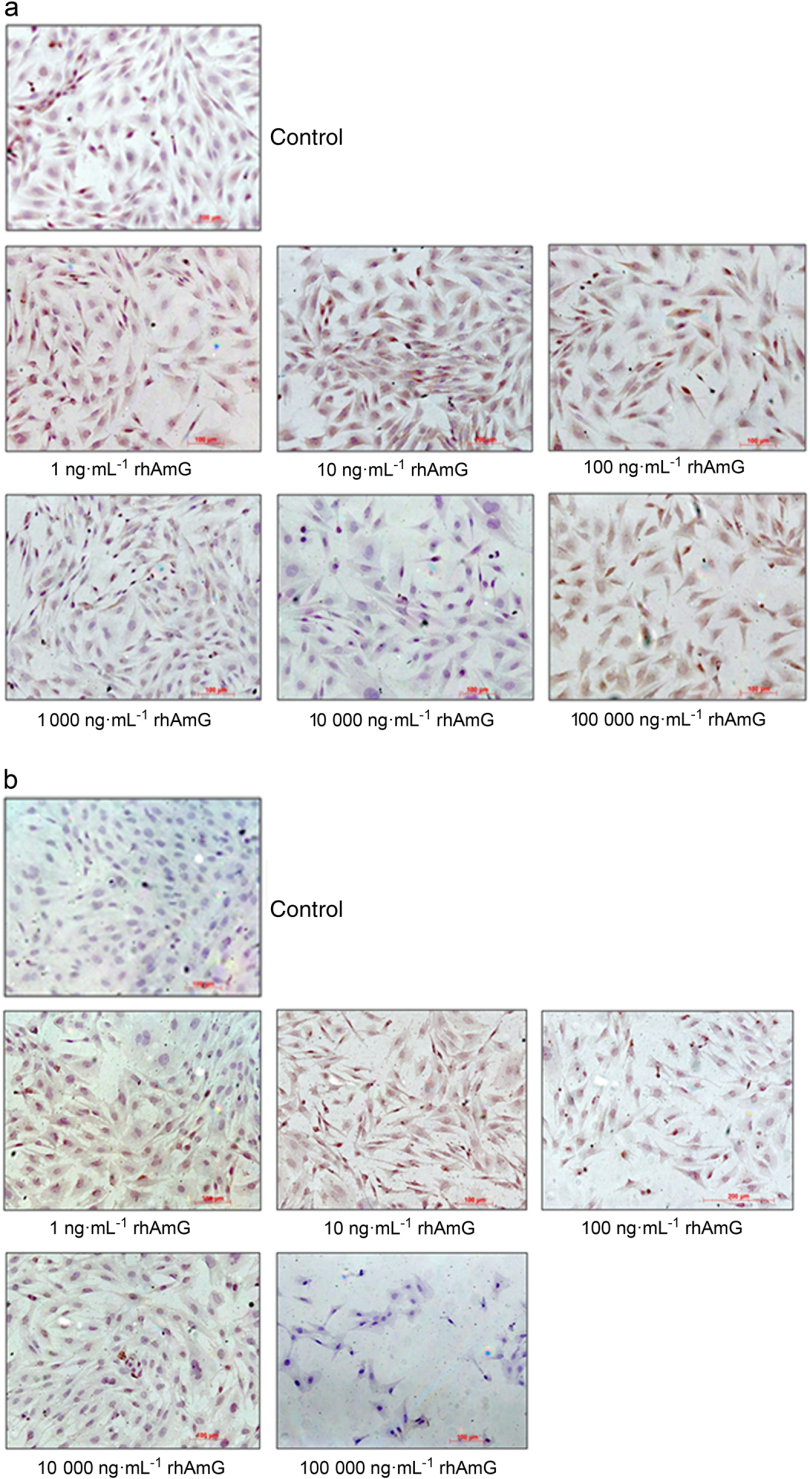

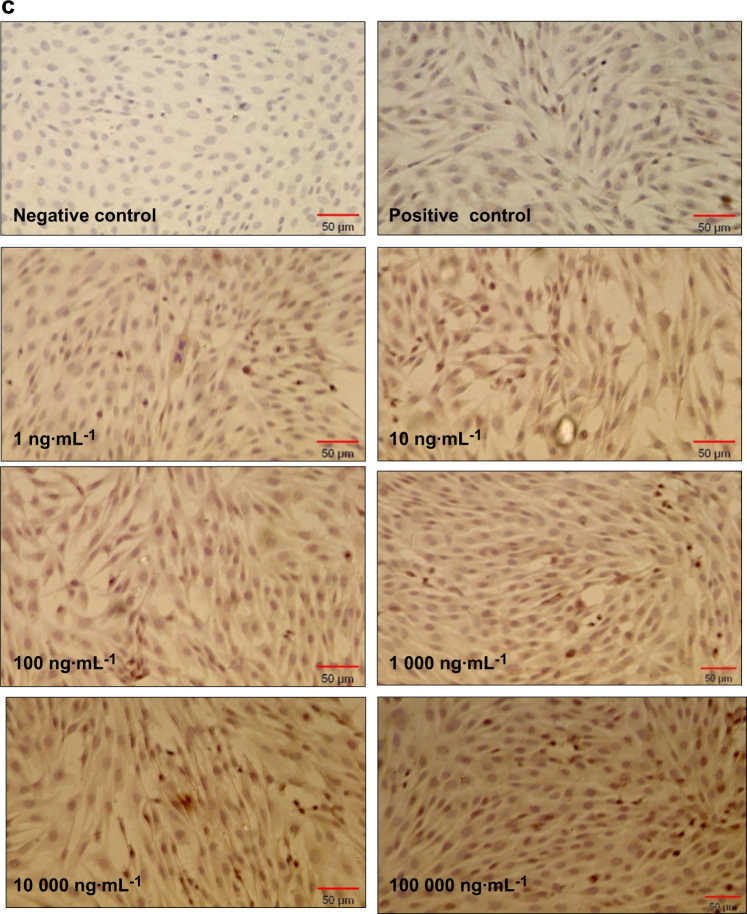
Effects of different rhAMG concentrations on **a** OPN, **b** COL I, and **c** LAMP-1 immunostaining of OCCM-30 cells; DAB, ×10

A subpopulation of non-stimulated cementoblasts exhibited weak to moderate cytoplasmic immunostaining, and other cells were negative (Fig. 4a). The intensity of staining increased in cells stimulated with 10 ng · mL^-1^ rhAMG, and higher concentrations exhibited a similar pattern for all concentrations tested.

#### Collagen type I

Few non-stimulated cells exhibited weak cytoplasmic immunostaining, and the remaining cells were not stained. Moderate staining in an increased number of cells was observed in cultures stimulated with 1–1000 ng · mL^-1^ (Fig. 4b), but higher concentrations produced an immunostaining pattern similar to that of non-stimulated cells.

#### LAMP-1

Non-stimulated cells and cells stimulated with 1, 10, 100, and 1000 ng rhAMG revealed no immunostaining (Fig. 4c). Few cells exhibited granular cytoplasmic immunostaining after stimulation with 10,000 ng, and nearly all cells were stained in the 100,000 ng · mL^-1^ rhAMG group.

### Immunofluorescence

**Fig. 5:**
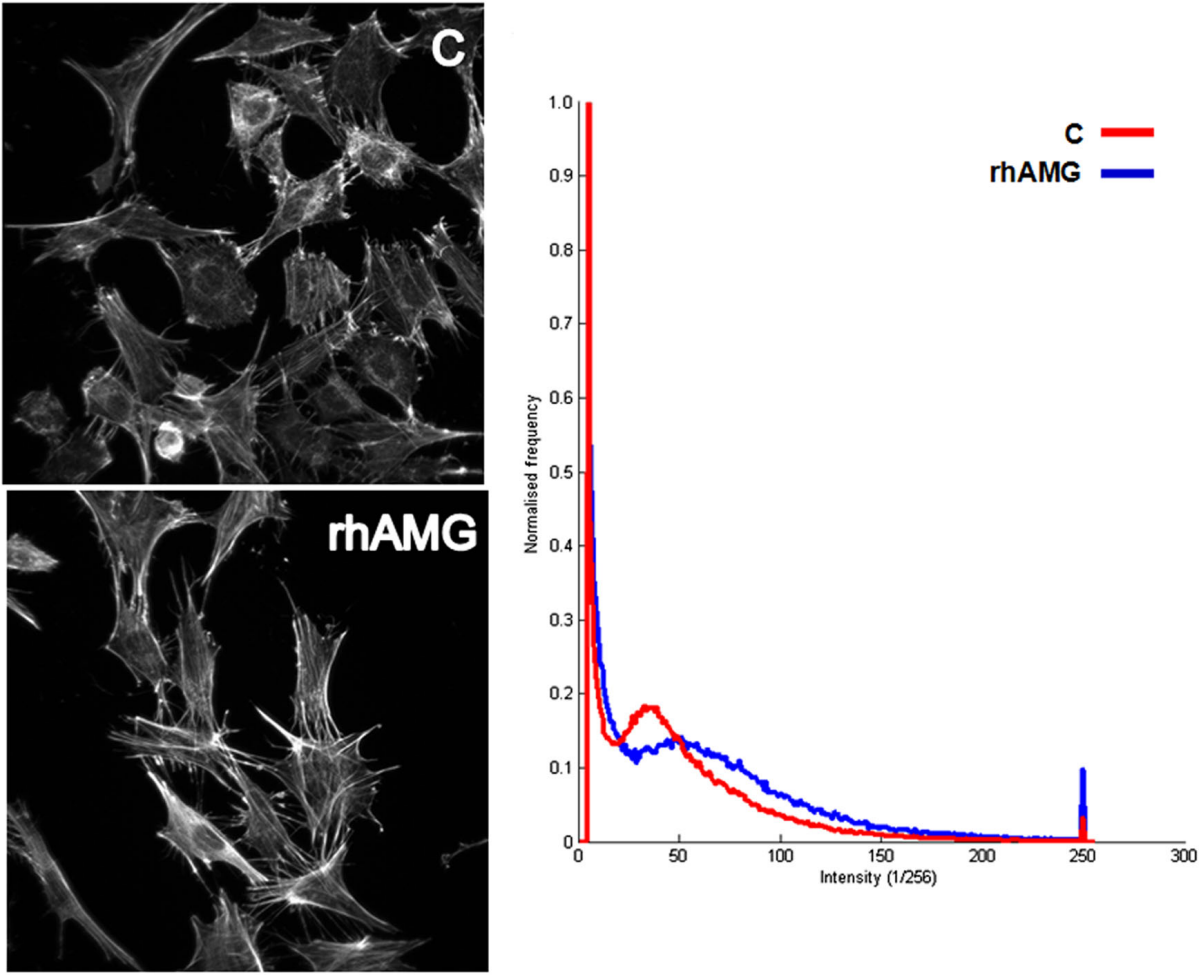
Effects of rhAMG on f-actin labeling. Projection images of OCCM cells incubated in control and 100 µg · mL^-1^ rhAMG-containing medium for 24 h. Alexa 488-conjugated phalloidin probe was used for fluorescent labeling. Amplitude histograms of the images are shown in left panel. C control, rhAMG recombinant human amelogenin

The fluorescence intensity of f-actin-labeled cementoblasts was slightly stronger with 100,000 rhAMG than with the control group (Fig. 5). rhAMG concentrations <100 µg · mL^-1^ induced no detectable change in fluorescence intensity. However, the fluorescence intensity was greater in cells incubated in 100 µg · mL^-1^ rhAMG. Intensity histograms shifted towards larger intensity amplitudes in rhAMG-treated cells than in the control group. No differences between experimental groups were observed for β1 integrin-labeled images.

## Discussion

Cementoblasts and their precursors have important roles in periodontal repair and regeneration via the formation of new cementum after proliferation and migration to the root surface.^37^ The OCCM-30 cells used in this study are immortalized mouse cementoblasts that are well characterized and widely used for cementoblast stimulation in vitro to investigate cementum regeneration.^38^ In this study, these cells were stimulated by a newly developed rhAMG with unknown biological functions on periodontium cells.^35^ We demonstrated that higher concentrations (e.g., 100,000 ng · mL^-1^) of rhAMG-stimulated cementoblast proliferation and mineralization and upregulated important osteogenic factors, such as ALP, BSP, Runx2, OCN, type I collagen, and OPN and the cementum-specific marker CAP.

RhAMG increased OCCM-30 cell proliferation. Murine cementoblasts exhibited enhanced cell proliferation after EMD exposure,^39,40^ but stimulation with a full-length murine AMG protein, an N-terminal AMG peptide or a leucine-rich AMP peptide (LRAP) produced no effects.^35,40,41^ Therefore, the rhAMG used in the present study may increase cementoblast proliferation. Induction of the proliferation of human cementoblast cell lines was demonstrated previously after treatment with another type of rhAMG.^42,43^ However, lower AMG concentrations were sufficient in these studies. Obviously, higher concentrations of AMG are required to stimulate proliferation in mouse cementoblasts. Previous studies failed to promote proliferation in mouse cementoblasts after treatment with concentrations up to 1000 ng · mL^-1^.^24^ Swanson et al.^24^ used 0.1, 1, and 10 µg · mL^-1^ tyrosine-rich amelogenin peptide (TRAP) on the same cementoblasts used in our study, and they observed no significant difference in cell proliferation among these three TRAP concentrations. Higher TRAP concentrations may have produced an effect on the cells. These cells are immortalized mouse cells, which may behave differently from primary human or mouse cells. The mitotic activity of OCCM-30 cells is higher than that of primary cells, and these cells may require higher AMG concentrations to induce cell functions.^24^

The rhAMG used in this study promoted mineralization in OCCM-30 cells as demonstrated by the mineralization assay. This result contrasts the stimulation of these cells with EMD, full-length murine AMG, LRAP and a tyrosine-rich AMG peptide, which produced no effects.^35,39–41^ Viswanathan et al.^40^ demonstrated decreased OCN and BSP mRNA transcripts and mineral nodule formation in vitro following treatment of mouse cementoblasts with a high dose (10 µg · mL^-1^) of full-length murine AMG protein (rp(H)M180). They observed similar cell proliferation in AMG-treated cells as the control. The present study revealed increased proliferation of mouse cementoblasts after treatment with a higher dose (100 µg · mL^-1^) that had not been used by Visvanathan et al.^40^ We also did not observe any significant changes at the other concentrations. In contrast, we observed increased ALP, OCN, and BSP expression levels and stronger biomineralization in cementoblasts. These differences may be explained by the use of murine AMG for stimulation in the Visvanathan study.^40^

Tanimoto et al.^44^ recently demonstrated that treatment of human cementoblasts (HCEMs) with recombinant human full-length AMG (rh174) upregulated ALP, OCN, and BSP mRNA levels. OCN and BSP protein levels, ALP activity and calcium deposition resulted in enhanced mineralization. These investigators used 0 to 1000 ng · mL^-1^ rh174. Similar results using a concentration of 1000 ng · mL^-1^ in our study demonstrated that murine cementoblasts required higher concentrations to achieve these effects.

Interestingly, similar results were observed when the same cell type was treated with LRAP or TRAP (tyrosine-rich amelogenin peptide, which is a degradation product of full-length AMG).^35,41^ The splice product LRAP has been shown to enhance the expression of Runx2, which is the most important factor for osteogenic differentiation.^45^ LRAP treatment induced a significant increase in mineral matrix formation and BSP in wild-type and AMG-null mouse embryonic stem cells.^46^ Amin et al.^17^ recently demonstrated that TRAP suppressed the formation of bone-like mineralized nodules, and LRAP upregulated osteogenic differentiation in bone precursor cells. These studies demonstrated that AMG and its peptides modulated the expression of mineralized tissue-associated genes and mineralization.^4^ The effects on mineralization are consistent with previous studies, which demonstrated the effects of EMD and AMG on osteoblasts, osteoblast-like cells, and stem cells.^47–52^ These data suggest a close relationship among cementoblasts, osteoblasts, osteoblast precursor cells, and mesenchymal stem cells.^37^ In contrast, PDL fibroblasts, which exhibit an osteogenic phenotype, did not upregulate mineralization markers after rhAMG treatment in one study.^44^

The expression data were only partially mirrored on the protein level in our study. OPN immunostaining also increased after stimulation with higher rhAMG concentrations, and type I collagen immunostaining was stronger only after stimulation with moderate concentrations. Tanimoto et al.^44^ demonstrated that recombinant AMG rh174 did not alter protein levels of OCN and BSP in human cementoblasts. No clear correlations between OPN and COLI expression levels and immunostaining intensity were observed. However, the control mechanisms for gene expression and translation in cementoblasts are not known. Epigenetic mechanisms driven by microRNAs may control expression in cementoblasts similarly to osteoblasts^53^ and influence protein secretion. The lack of evaluation of mineralized tissue/cementum-associated protein (BSP and OCN) levels was a limitation of this study. Quantification of protein levels would substantially improve our data and confirm the changes in mRNA expression in the OCCM-30 cells treated with rhAMG.

RhAMG-induced CAP upregulation in mouse cementoblasts may be interpreted in the circumstance of wound healing and cementum repair and regeneration because CAP has a regulatory role in cementum formation and the promotion of attachment of PDL cells, gingival fibroblasts, and endothelial cells. CAP is also involved in the promotion of the differentiation of cementoblast precursors.^54^

Our results demonstrated for the first time that OCCM-30 cells express LAMP-1, which is a putative AMG receptor, after rhAMG stimulation. The results of our immunohistochemical experiments demonstrated that stimulated LAMP-1 staining was observed in the 100,000 ng · mL^-1^ rhAMG group. LAMP-1 may be a cell surface receptor for the specific amelogenin isoform leucine-rich amelogenin peptide. Zhang et al.^55^ used full-length recombinant mouse amelogenin, rp(H)M180, on murine dental follicle cells and cementoblasts (OCCM-30) and observed maximum surface binding at 50 µg · mL^-1^ of rp(H)M180. Their data suggested that LAMP-1 is a cell surface binding site for amelogenin on dental follicle cells and cementoblasts. Tanimoto et al.^44^ also suggested that LAMP-1 signaling was responsible for the effects of rh174 AMG on the mineralization of human cementoblasts. However, Kunimatsu et al.^43^ demonstrated that the cell surface antigen CD63 interacted with rhAMG.

The present results revealed no effects on integrin immunostaining after rhAMG stimulation. This observation suggests that rhAMG treatment does not influence cell adhesion at the concentrations used in this study. This result contrasts observations in human dermal fibroblasts in which AMG increased the binding of these cells via several integrins.^56^ Only weak effects were observed for actin immunostaining, which suggests a weak influence on the migration of OCCM-30 cells. In contrast, EMD induced lymphocyte migration in a dose-dependent manner.^57^ Figure 5 shows that the fluorescence intensity increased in the presence of 100 µg · mL^-1^ rhAMG. Histogram analyses indicated greater intensities at a higher frequency in the 100 µg · mL^-1^ rhAMG-treated group. Therefore, we suggest that rhAMG (100 µg · mL^-1^) treatment induced an increase in the f-actin content of cementoblast cells. Notably, the cells in our study were incubated, labeled and imaged under identical conditions, and normalized histogram analyses yielded a reliable quantitative parameter that was independent of the number of cells in the imaged frame. The fluorescent labeling and confocal microscopy results support the PCR and proliferation experiments.

Defining the roles of different types of AMG (i.e., murine, human, recombinant) and its peptides in modulating the activity of cementoblasts or progenitor cells in vitro will provide critical information for the design of regenerative periodontal therapies. Further comparative studies will reveal whether the rhAMG examined in this study is superior to natural EMD, AMG or its peptides in cementoblasts. Notably, rhAMG may be used as a fusion partner between other proteins that may be important for regeneration, e.g., PTH or BMPs.^58,59^ Functionalized rhAMG may also have a role in tissue engineering in the future.^60^

## Materials and methods

An immortalized cementoblast cell line (OCCM-30) was used for these studies. The methods for isolating and immortalizing these cells have been published previously.^38,61^ Cells were kindly provided by Dr. Martha J. Somerman (NIDCR, NIH, USA). rhAMG was kindly donated by Johan Svensson (Department of Pure and Applied Biochemistry, Center for Chemistry and Chemical Engineering, Lund University, Lund, Sweden)

### Preparation of recombinant AMG

Briefly, a gene encoding the X-chromosomal human 175 amino acid amelogenin (NCBI accession number AAA51717, excluding the 16 amino acid N-terminal signal peptide) was synthesized using PCR. Nine oligonucleotides ~80 bp long with ~20 bp complementary ends were used to construct the gene, which was codon optimized for the *E. coli* expression strain BL21 (DE3). The oligonucleotides were mixed and assembled using PfuUltra Hotstart DNA polymerase (Stratagene). The assembled amelogenin gene was amplified using PCR with flanking primers and the assembly mixture as templates. The gene was subsequently cloned into a cloning vector and sequenced. Point mutations were corrected using a QuickChange Site-Directed Mutagenesis Kit (Stratagene). The gene was inserted between the *Nde*I and *Bam*HI sites in the expression vector pET11a (Novagen). Purification of the prokaryotic cell suspension was performed, and the supernatant containing the soluble fraction of amelogenin was analyzed by SDS-PAGE, HPLC, and MS.^35,58^

### Cell culture

Cells were allowed to adhere for 24 h in Dulbecco’s modified Eagle’s medium (DMEM) with 10% foetal bovine serum (FBS). The medium was changed to DMEM^††^ with 5% FBS containing different concentrations of AMG. The cells used in these experiments were between passages 19 and 21. Experiments were performed twice in triplicate for each experiment for RNA isolation and three times in triplicate for mineralization.

### rhAMG stimulation and real-time cell analysis

OCCM-30 cells were treated with DMEM containing 5% FBS and different concentrations of rhAMG (0.1, 1, 10, 100, 1000, 10,000, and 100,000 ng · mL^-1^). Five percent FBS was used as control. OCCM-30 cell proliferation was investigated using real-time cell analysis in an RTAC analyzer (RT-CES; xCELLigence, CEA Biosciences, Inc., San Diego, CA, USA). Real-time and continuous monitoring allows label-free assessment of cell proliferation, viability, and cytotoxicity, and reveals the physiological state of the cells. Cells attached to the electrode sensor surfaces act as insulators and thereby alter the local ion environment at the electrode-solution interface, which increases impedance. Therefore, the increased numbers of cells growing on the electrodes increases electrode impedance. Cell suspensions (200 µL) were seeded into the wells (5000 cells/well) of an E-plate 96 (ACEA Biosciences), and OCCM-30 cells were monitored every 15 min for 144 h.

### RNA isolation

Cells were allowed to adhere for 24 h in DMEM with 10% FBS, and media were changed to DMEM with 5% FBS containing different concentrations of rhAMG. To determine gene expression, OCCM-30 cells were plated in 60-mm cell culture dishes (Corning, New York, USA) at 5 × 10^4^ cells/cm^2^ and treated after 24 h as follows: (1) 5% FBS (as control), (2) 5% FBS + 0.1 ng · mL^-1^ rhAMG, (3) 5% FBS + 1 ng · mL^-1^ rhAMG, (4) 5% FBS + 10 ng · mL^-1^ rhAMG, (5) 5% FBS + 100 ng · mL^-1^ rhAMG, (6) 5% FBS + 1000 ng · mL^-1^ rhAMG, (7) 5% FBS + 10,000 ng · mL^-1^ rhAMG, and (8) 5% FBS + 100,000 ng · mL^-1^ rhAMG. Total RNA was isolated 72 h after treatment using a monophasic solution of phenol and guanidine isothiocyanate (InVitrogen, Camarillo, CA, USA). RNA concentrations were quantified at 260 nm using a spectrophotometer (Nanodrop, Wilmington, DE, USA), and RNA samples were stored at −80 °C.

### cDNA synthesis and real-time quantitative RT-PCR

For real-time RT-PCR analysis, cDNA was synthesized from 1.0 µg of total RNA using a cDNA synthesis kit (High Capacity RNA-to-cDNA kit, Applied Biosystems, Foster City, Carlsbad, California, USA). A volume of 1.0 µL of the resulting cDNA product was used per 25 µL final reaction volume in a thermal cycler (Stratagene MX3000P, La Jolla, California, USA). PCR was performed using a Brilliant SYBR Green QPCR Master Mix kit (Stratagene, La Jolla, California, USA) in a total volume of 25 µL. Primers were designed using DNA-Star design software. Table 1 lists the sequences. A BLAST search of GenBank was performed on the primer sequences to ensure specificity. GAPDH served as a housekeeping/reference gene for normalization. The amplification profiles of OCN, Runx2, COL I, and GAPDH used for the Stratagene MX3000P were 94/180; 94/45, 54/45, 95/60, 55/30, and 95/30 [temperature (°C)/time (s)], respectively, for 35–40 cycles. The amplification profiles for BSP, OPN, and GAPDH used for the Stratagene MX3000P were 94/180; 94/45, 52/45; 95/60, 55/30, and 95/30 [temperature (°C)/time (s)], respectively, for 35–40 cycles. The amplification profiles for CAP, ALP, and GAPDH used for the Stratagene MX3000P were 95/600; 95/15, 60/60; 95/60, 55/30, and 95/30 [temperature (°C)/time (s)], respectively, for 35–40 cycles. Quantitative RT-PCR experiments were repeated three times.

**Table 1:** Primer sequences for mineralized tissue-associated genes for mouse

Primer	Forward	Reverse
COL I	GCAACATTGGATTCCCTGGACC	GTTCACCCTTTTCTCCCTTGCC
BSP	GAGACGGCGATAGTTCC	AGTGCCGCTAACTCAA
OCN	TGAACAGACTCCGGCG	GATACCGTAGATGCGTTTG
OPN	TTTACAGCCTGCACCC	CTAGCAGTGACGGTCT
Runx2	CTTCATTCGCCTCACAAAC	GTCACTGCGCTGAAGA
CAP	TCTGACGACTCTGCTTCACG	TTCAGGGCATGTGTGATGCT
ALP	ATTGCCCTGAAACTCCAAAACC	CCTCTGGTGGCATCTCGTTATC
GAPDH	ACCACAGTCCATGCCATCAC	TCCACCACCCTGTTGCTGTA

### Mineralization assay

The cells used in these experiments were between passages 19 and 21. Cells were plated at 5 × 10^4^ cells/cm^2^ in 24-well plates in DMEM containing 10% FBS for 24 h and exposed to the following factors: (a) 5% FBS + β-glycerophosphate (10 mM), (b) 5% FBS + Mineralization Media [MM = ascorbic acid (AA, 50 μg/ml) and β-glycerophosphate (BGP, 10 mM)], (c) 5% FBS + rhAMG (0.1 ng · mL^-1^) + MM, (d) 5% FBS + rhAMG (1 ng · mL^-1^) + MM, (e) 5% FBS + rhAMG (10 ng · mL^-1^) + MM, (f) 5% FBS + rhAMG (100 ng · mL^-1^) + MM, (g) 5% FBS + rhAMG (1000 ng · mL^-1^) + MM, (h) 5% FBS + rhAMG (10,000 ng · mL^-1^) + MM, and (i) 5% FBS + rhAMG (100,000 ng · mL^-1^) + MM. Mineralization of extracellular matrix was determined on day 8 by von Kossa staining. Briefly, cells were washed with PBS twice, fixed with 100% ethanol at 37 °C for 1 h and washed in a descending alcohol series (90 to 50%) to deionized water. Cells were treated with 5% AgNO_3_ and incubated at 37 °C in the dark for 15 min and washed with deionized water. Plates were exposed to fluorescent light for 20 min, and photographs were obtained.^59^

### Immunostaining

Cementoblasts were cultured on sterile coverslips for 48 h and fixed in 4% paraformaldehyde for 15 min. Cells were washed in PBS und subsequently permeabilized with 0.05% Triton X-100 (Sigma) in PBS. The cells were washed and treated with 2% goat serum (Dako, Glostrup, Denmark) for 1 h at room temperature to reduce non-specific binding. The following primary antibodies were incubated for 4 h at 37 °C: rabbit polyclonal anti-osteopontin (1:200; Abcam, Cambridge, USA), mouse monoclonal anti-collagen type I (1:200; Abcam), and mouse monoclonal anti-LAMP (1:500; Abcam). Peroxidase-conjugated anti-mouse or anti-rabbit EnVision® (Dako) secondary antibodies were incubated for 30 min at room temperature. Diaminobenzidine (DAB) was used as the substrate. Cells were counterstained with haemalaunum. Primary and secondary antibodies were omitted in the negative controls.

### Confocal microscopy

Cells were examined using a confocal laser scanning microscope (CFLSM) to observe the effects of rhAMG on cell adhesion. Cell viability, successful growth and proliferation in the new environment were determined. Cells were fluorescently labeled using lipophilic dye Dil as described in Hakki et al.^62^ OCCM-30 cells were seeded at an equal density (30 × 10^3^/mL) in cover glass bottom multi-well plates. Half of the wells were filled with control medium, and the other wells contained various rhAMG solutions (in control medium). Cells were fixed at 37^o^C in 5% CO_2_ for 24 h, and actin bundles were fluorescently labeled.

For imaging f-actin, cells were fixed in 4% PFA, permeabilized, and blocked in 1% BSA. Samples were washed several times in PBS and incubated in an Alexa 488-labeled phalloidin solution for 30 min at room temperature (5 U/200 μL PBS). Samples were washed and mounted in an antifade solution. Plates were mounted on a confocal microscope, and cells were imaged under the same recording conditions. The 488 nm line of an argon ion laser was used for excitation, and emissions were collected using an LP 510 nm barrier filter. *Z*-stack images were acquired, and 3D projection images were constructed for analyses. For β1 integrin imaging, cells were fixed, permeabilized and blocked in 1% goat serum. Samples were incubated in primary antibodies raised against human integrin in mouse for 12–24 h in PBS. Samples were washed several times in PBS and incubated with an Alexa 488-labeled goat anti-mouse secondary antibody. Samples were washed and mounted in an antifade solution. The 488 nm line of an argon ion laser was used for excitation, and emissions were collected through an LP 510 nm barrier filter. *Z*-stack images were acquired, and 3D images were constructed for topographical analyses of cell growth and quantification. Recordings were analyzed to observe changes in fluorescent labeling between test groups and the control group. Amplitude histograms were constructed from the projection images. The first lowest intensity levels were ignored to eliminate background labeling.

### Statistical analysis

Proliferation and RT-PCR results were analyzed using one-way analysis of variance (ANOVA) and Tukey–Kramer multiple comparison tests. Data are presented as the means ± standard deviation.
